# Supplementary material for: Meta-analysis of (single-cell method) benchmarks reveals the need for extensibility and interoperability
Source: Genome Biol. 2023 May 17;24:119. doi: 10.1186/s13059-023-02962-5 (PMC10189979; doi:10.1186/s13059-023-02962-5)
Supplement: Supplementary file 4 — Additional file 4: Figure S1. Auxiliary summaries from the survey responses. Simulator used, secondary measures assessed, language of tools assessed. [file 13059_2023_2962_MOESM4_ESM.pdf]

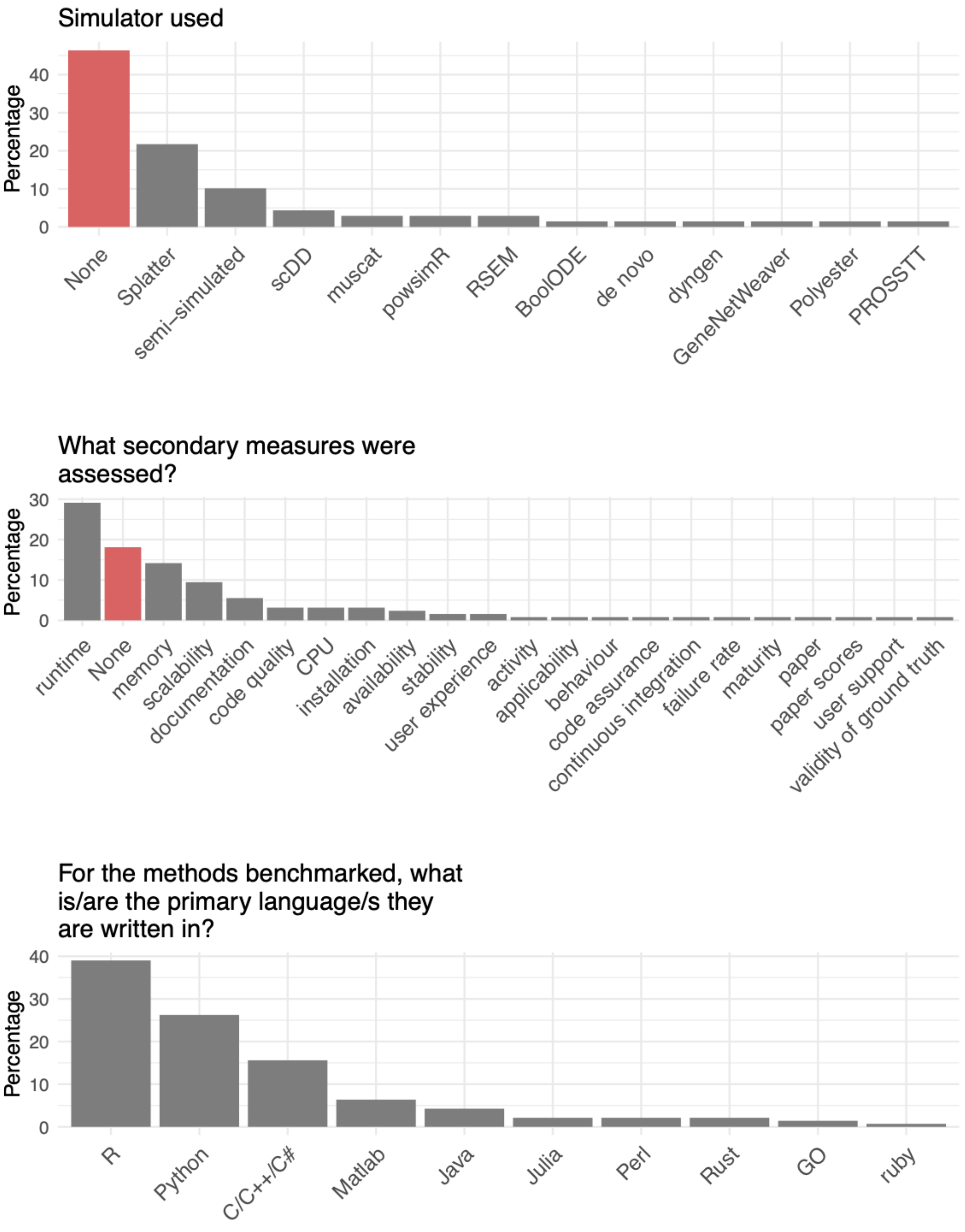

Figure S1. **Auxiliary summaries from the survey responses**. Simulator used, secondary measures assessed, language of tools assessed.
