# Supplementary material for: Meta-analysis of (single-cell method) benchmarks reveals the need for extensibility and interoperability
Source: Genome Biol. 2023 May 17;24:119. doi: 10.1186/s13059-023-02962-5 (PMC10189979; doi:10.1186/s13059-023-02962-5)
Supplement: Supplementary file 5 — Additional file 5. Review history. [file 13059_2023_2962_MOESM5_ESM.docx]

**First round of review**

**Reviewer 1**

**Were you able to assess all statistics in the manuscript, including the appropriateness of statistical tests used?** Yes, and I have assessed the statistics in my report.

**Comments to author:**

The single-cell technology is being developed in an unprecedented speed, and so many new methods are been proposed every month. Every method claims its own superiority over existing methods, but it's almost impossible for users and researchers in this field to know which methods are truly better. This highlights the importance of review and benchmark papers, which compare multiple methods on multiple datasets. However, even these benchmark papers themselves have different qualities, at least in the sense of extensibility and interoperability. This paper discusses about the extensibility and interoperability of benchmarks based on a survey they conducted. Thus, without doubt, this paper is focusing on a crucial and urgently needed topic. I had a high expectation when I read the title and the abstract. However, the content of the paper turns out to be quite disappointing because of lacking of novel discoveries and of insightful and practical advices. I do not feel that I have learnt much from the paper or the paper can be much helpful to improving the quality of benchmarks in the field.

1. While focusing on the cutting-edge field--single-cell methods--the observations on the benchmarks are nothing much different from benchmarks in other fields. I was not surprised by any of the observations in the "Code / data availability, reproducibility and technical aspects" section. This means that readers of the paper do not really get much new information.

2. Their suggestions regarding how to improve the quality of benchmarks in this field are also not novel, and no concrete guidelines are given to make them practical. For example, "A better standard would involve updating and running benchmarking continuously as new methods emerge and importantly." Of course, this would be great, but how to make this happen? Another example is the suggestion of using "containerization," which is a well-known solution for improving code reusability and extensibility in science and engineering in general, but any concrete suggestions on how to reinforce this? This means that the journal publishers may not be able to get much from this paper to set guidelines/rules to improve extensibility and interoperability of benchmarks to the single-cell field.

Further, I find some conclusions/suggestions made by this paper are not well supported by their survey. For example, "Taken together, our meta-analysis shows that most benchmarks results are at least in principle reproducible, since code and data are shared. However, extending or updating benchmarks remains challenging because setting up software environments locally is challenging and intermediate results are not available." How is the "However, ..." sentence supported by the previous observation?

**Authors Response**

**Point-by-point responses to the reviewers’ comments:**

*Reviewer #1: The single-cell technology is being developed in an unprecedented speed, and so many new methods are been proposed every month. Every method claims its own superiority over existing methods, but it's almost impossible for users and researchers in this field to know which methods are truly better. This highlights the importance of review and benchmark papers, which compare multiple methods on multiple datasets. However, even these benchmark papers themselves have different qualities, at least in the sense of extensibility and interoperability. This paper discusses about the extensibility and interoperability of benchmarks based on a survey they conducted. Thus, without doubt, this paper is focusing on a crucial and urgently needed topic. I had a high expectation when I read the title and the abstract. However, the content of the paper turns out to be quite disappointing because of lacking of novel discoveries and of insightful and practical advices. I do not feel that I have learnt much from the paper or the paper can be much helpful to improving the quality of benchmarks in the field.*

1. *While focusing on the cutting-edge field--single-cell methods--the observations on the benchmarks are nothing much different from benchmarks in other fields. I was not surprised by any of the observations in the "Code / data availability, reproducibility and technical aspects" section. This means that readers of the paper do not really get much new information.*

Our findings may indeed not be surprising for some readers, but the goal here is not the novelty of results, but rather to establish hard numbers on a modern area of method explosion. We are reporting multiple issues that were also found in other subfields of life science and bioinformatics (Radhouene et al. 2010 doi: [10.1093/nar/gkq625](https://doi.org/10.1093%2Fnar%2Fgkq625), Feijoo et al. 2020 doi:[10.1007/978-3-030-65847-2_12](http://dx.doi.org/10.1007/978-3-030-65847-2_12)), but the results show that we as a community can achieve higher standards. Our primary goal was to assess these problems in the field of single-cell method development, where the rapid progress makes the need for good benchmarking even more crucial. The secondary goal of this meta-analysis was to try to quantify the advances of the community in adopting FAIR principles (e.g. 75% do report their code) and the areas where these principles are still lacking (e.g., use of workflow system and containerization). Lastly, we hope that by pinpointing the “less-good” practices, we can highlight areas of improvements for future efforts of benchmarking.

2. *Their suggestions regarding how to improve the quality of benchmarks in this field are also not novel, and no concrete guidelines are given to make them practical. For example, "A better standard would involve updating and running benchmarking continuously as new methods emerge and importantly." Of course, this would be great, but how to make this happen? Another example is the suggestion of using*

*"containerization," which is a well-known solution for improving code reusability and extensibility in science and engineering in general, but any concrete suggestions on how to reinforce this? This means that the journal publishers may not be able to get much from this paper to set guidelines/rules to improve extensibility and interoperability of benchmarks to the single-cell field.*

We thank the reviewer for the comment regarding the lack of clear guidelines. In the first version, we have opened a discussion on the pitfalls and the possible solutions that we envision for future benchmarking efforts (‘The future of benchmarking’ section). Our work is mostly descriptive and aims to quantify the current state of benchmarking and several guidelines have already been published (e.g. Weber et al 2019 <https://pubmed.ncbi.nlm.nih.gov/31221194/> and Radhouene et al. 2010 <https://www.ncbi.nlm.nih.gov/pmc/articles/PMC2995051/>). However, we agree that our thoughts on strategies to improve the current practices could be more clearly defined and could benefit from a clear set of rules.

To make our guidelines more clear, we have added a table of suggestions (Table 1) that defines the main areas of improvements and our suggestions (summarized from our observations in the Results and from our ideas in the Discussion section). We now cite this table, and the previously published guidelines, in the very last paragraph of our manuscript.

Further, I find some conclusions/suggestions made by this paper are not well supported by their survey. For example, "Taken together, our meta-analysis shows that most benchmarks results are at least in principle reproducible, since code and data are shared. However, extending or updating benchmarks remains challenging because setting up software environments locally is challenging and intermediate results are not available." How is the "However, ..." sentence supported by the previous observation?

The sentence identified by the reviewer could indeed be clarified and we thank the reviewer for noticing it. We have modified this text to be clearer and have a more logical sequence to emphasize that surveyed benchmarks do not meet all requirements for reproducibility, despite providing code and input data.
